# The complete chloroplast genome sequence of *Litsea cubeba*

**DOI:** 10.1080/23802359.2020.1768961

**Published:** 2020-05-27

**Authors:** Qinghua Wang, Huangyijun Wang, Yunqin Li, Xiaolong Yuan, Ting Luo, Yi Wang

**Affiliations:** a Laboratory of Forest Plant Cultivation and Utilization, Yunnan Academy of Forestry & Grassland Science, Kunming, Yunnan, China; b College of Forestry, Fujian Agriculture and Forestry University, Fuzhou, Fujian, China

**Keywords:** *Litsea cubeba*, chloroplast, Illumina sequencing, phylogenetic analysis

## Abstract

The first complete chloroplast genome (cpDNA) sequence of *Litsea cubeba* was determined from Illumina HiSeq pair-end sequencing data in this study. The cpDNA is 152,725 bp in length, contains a large single-copy region (LSC) of 93,673 bp, and a small single-copy region (SSC) of 18,924 bp, which were separated by a pair of inverted repeats (IR) regions of 20,064 bp, each. The genome contains 126 genes, including 82 protein-coding genes, 8 ribosomal RNA genes, and 36 transfer RNA genes. The further phylogenomic analysis showed that *L. cubeba and Litsea garrettii* clustered in a clade in Lauraceae family.

*Litsea cubeba* is the species of the genus *Litsea* within the family Lauraceae. It grows wildly in southeast Asian countries, including India, China, Bhutan, Nepal, Myanmar, Vietnam, Korea, Taiwan and Indonesia (Bhuinya et al. 2010). The flowers, leaves, and roots of *L. cubeba* are used for flavoring. Its fruit is edible and the dried fruits are used in traditional Chinese medicine (Yang et al. 2014). The essential oil of *L. cubeba* also showed the antibacterial, antioxidant, and antiparasitic activity, it also showed genetic toxicity, cytotoxicity and was a potential cancer prevention agent (Wang et al. 2012; Huang et al. 2013). However, there have been no genomic studies on *L. cubeba.*

Herein, we reported and characterized the complete *L. cubeba* plastid genome. The GenBank accession number is MT431385. One *L. cubeba* individual (specimen number: 2020007) was collected from Kunming, Yunnan Province of China (25°5′11′′N, 102°40′40′′E). The specimen is stored at Yunnan Academy of Forestry Herbarium, Kunming, China and the accession number is WQH002. DNA was extracted from its fresh leaves using DNA Plantzol Reagent (Invitrogen, Carlsbad, CA).

Paired-end reads were sequenced by using Illumina HiSeq system (Illumina, San Diego, CA). In total, about 21.7 million high-quality clean reads were generated with adaptors trimmed. Aligning, assembly, and annotation were conducted by CLC de novo assembler (CLC Bio, Aarhus, Denmark), BLAST, GeSeq (Tillich et al. 2017), and GENEIOUS v11.0.5 (Biomatters Ltd., Auckland, New Zealand). To confirm the phylogenetic position of *L. cubeba*, the other 13 species of Lauraceae family from NCBI were aligned using MAFFT v.7 (Katoh and Standley 2013). The Auto algorithm in the MAFFT alignment software was used to align the sixteen complete genome sequences and the G-INS-i algorithm was used to align the partial complex sequences. The maximum-likelihood (ML) bootstrap analysis was conducted using RAxML (Stamatakis 2006); bootstrap probability values were calculated from 1000 replicates. *Chimonanthus nitens* (MH377058) and *Chimonanthus praecox* (MH377057) served as the out-group.

The complete *L. cubeba* plastid genome is a circular DNA molecule with the length of 152,725 bp, contains a large single-copy region (LSC) of 93,673 bp and a small single-copy region (SSC) of 18,924 bp, which were separated by a pair of inverted repeats (IR) regions of 20,064 bp, each. The overall GC content of the whole genome is 39.2%, and the corresponding values of the LSC, SSC, and IR regions are 38.0, 33.9, and 44.4, respectively. The plastid genome contained 126 genes, including 82 protein-coding genes, 8 ribosomal RNA genes, and 36 transfer RNA genes. Phylogenetic analysis showed that *L. cubeba*, *Litsea garrettii* and *Litsea glutinosa* clustered in a unique clade in Lauraceae family (Figure 1). The determination of the complete plastid genome sequences provided new molecular data to illuminate the Lauraceae family evolution.

**Figure 1.**
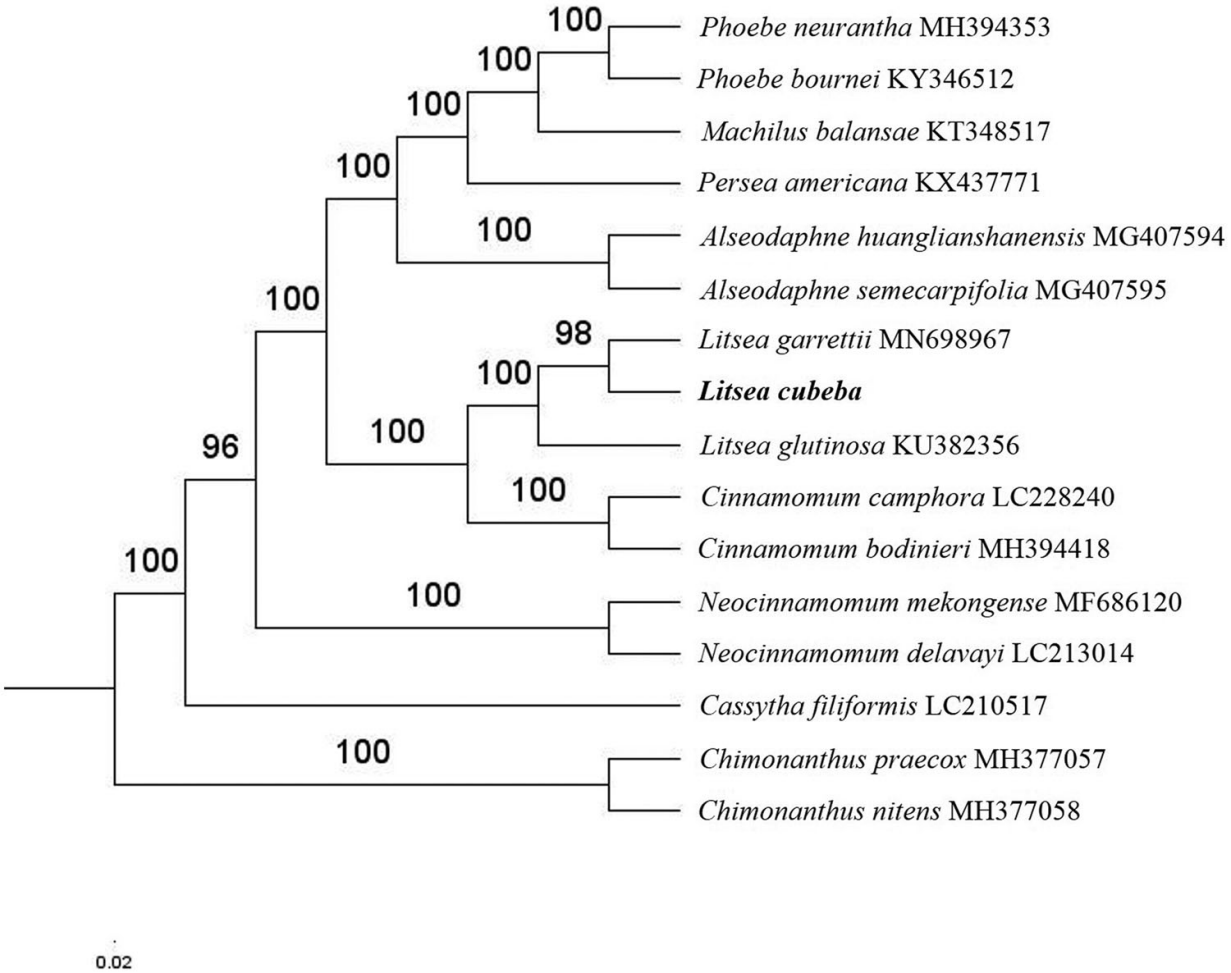
The maximum-likelihood tree based on the fourteen chloroplast genomes of Lauraceae family. The bootstrap value based on 1000 replicates is shown on each node.

## Data Availability

The data that support the findings of this study are openly available in NCBI GenBank database at (https://www.ncbi.nlm.nih.gov) with the accession number is MT431385, which permits unrestricted use, distribution, and reproduction in any medium, provided the original work is properly cited.

## References

[CIT0001] Bhuinya T, Singh P, Mukherjee SK. 2010. *Litsea cubeba* medicinal values brief summary. J Trop Med Plants. 11(2):179–183.

[CIT0002] Huang XW, Feng YC, Huang Y, Li HL. 2013. Potential cosmetic application of essential oil extracted from *Litsea cubeba* fruits from China. J Essent Oil Res. 25(2):112–119.

[CIT0003] Katoh K, Standley DM. 2013. MAFFT multiple sequence alignment software version 7: improvements in performance and usability. Mol Biol Evol. 30(4):772–780. 23329690 10.1093/molbev/mst010 PMC3603318

[CIT0004] Stamatakis A. 2006. RAxML-VI-HPC: maximum likelihood-based phylogenetic analyses with thousands of taxa and mixed models. Bioinformatics. 22(21):2688–2690. 16928733 10.1093/bioinformatics/btl446

[CIT0005] Tillich M, Lehwark P, Pellizzer T, Ulbricht-Jones ES, Fischer A, Bock R, Greiner S. 2017. GeSeq - versatile and accurate annotation of organelle genomes. Nucleic Acids Res. 45(W1):W6–W11. 28486635 10.1093/nar/gkx391 PMC5570176

[CIT0006] Wang Y, Jiang ZT, Li R. 2012. Antioxidant activity, free radical scavenging potential and chemical composition of *Litsea cubeba* essential oil. J Essent Oil Bear Plants. 15(1):134–143.

[CIT0007] Yang K, Wang CF, You CX, Geng ZF, Sun RQ, Guo SS, Du SS, Liu ZL, Deng ZW. 2014. Bioactivity of essential oil of *Litsea cubeba* from China and its main compounds against two stored product insects. J Asia Pac Entomol. 17(3):459–466.

